# Collective Science to Inform Global Ocean Protections

**DOI:** 10.1111/ele.70168

**Published:** 2025-08-07

**Authors:** William K. Oestreich, Max F. Czapanskiy, Kakani Katija, Nicholas R. Record, Melissa S. Chapman

**Affiliations:** ^1^ Monterey Bay Aquarium Research Institute Moss Landing California USA; ^2^ Bren School of Environmental Science & Management and the College of Creative Studies University of California Santa Barbara Santa Barbara California USA; ^3^ Tandy Center for Ocean Forecasting, Bigelow Laboratory for Ocean Sciences East Boothbay Maine USA; ^4^ Department of Environmental Systems Science ETH Zürich Zürich Switzerland

**Keywords:** area‐based conservation, areas beyond national jurisdiction, biodiversity, data governance, deep sea, high seas treaty, ocean, open science, participatory science, spatial conservation planning

## Abstract

In June 2023, the United Nations adopted the High Seas Treaty. Two years later, signatories are poised to ratify this treaty and create a legal instrument to implement marine protected areas (MPAs) in our greatest global commons, ocean areas beyond national jurisdiction. Protection of the open and deep ocean is timely: we stand at the precipice of an industrial revolution in Earth's largest remaining wilderness. Deciding where to strategically implement high seas MPAs under this treaty requires robust biodiversity information, yet publicly accessible data is sparse, particularly at depth. There is now an opportunity for collective science action to support this collective policy action. Realising this opportunity necessitates swift solutions including (1) supporting and incentivising standardised public sharing of existing biodiversity data; (2) broadening the scope of participatory science to process ocean observations into biodiversity data; and (3) equitably implementing new data collection with research partners across our global community.

## Graphical abstract

Signatories are poised to ratify the UN High Seas Treaty, providing a legal mechanism to create protected areas in our greatest global commons. Robust, publicly accessible biodiversity data is needed to enable effective and equitable proposals for protection under the treaty but is relatively scarce, particularly at depth. Through coordinated actions, we can collectively provide solutions to fill this biodiversity data gap and maximise the potential efficacy of protections under the High Seas Treaty.

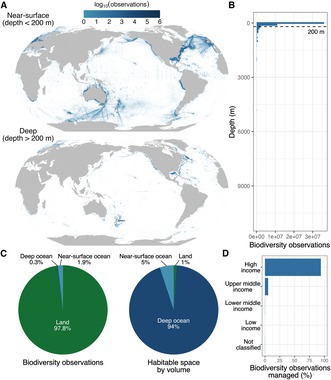

We live on an ocean planet: oceanic ecosystems represent 99% of the Earth surface's habitable volume, with deep‐ocean ecosystems (i.e., waters and benthos > 200 m deep) alone comprising 94% of this space (Haddock and Choy 2024). This vast expanse is home to diverse and abundant lifeforms which play central roles in ecosystem functioning, global nutrient cycling and climate regulation (Robison 2009; Robison et al. 2005). Yet the ocean's abundance of space, energy and life are also extraordinary natural resources, many of which have historically remained beyond humanity's industrial reach. Past and present industrial impacts (e.g., fisheries, energy extraction, shipping and climate change) represent just the tip of the iceberg: the scale and scope of industrial activity is rapidly accelerating throughout the water column and seafloor. Technological advances and increasing societal demands have opened the door to mesopelagic fisheries, offshore wind and tidal energy infrastructure and deep‐sea mining, with potentially deleterious effects on biodiversity (Drazen et al. 2020; Jacquemont et al. 2024).

With the International Seabed Authority's inability to reach an agreement for the regulation of deep‐sea mining, the High Seas Treaty (HST) represents society's remaining mechanism for delineating areas of the high seas to be protected from industrialisation. Specifically, the HST enables establishment of ‘ecologically representative and well‐connected networks of marine protected areas’, to be proposed ‘on the basis of the best available science and scientific information and, where available, relevant traditional knowledge of Indigenous Peoples and local communities’ (United Nations 2023). This objective raises important questions for our global community: What scientific information and traditional knowledge is *available* to inform such protections? How do information gaps limit our capacity to maximise the potential efficacy of area‐based protections? And how can we collectively provide critical biodiversity data to inform the use of this legal instrument?

## 1 Diving Deep for Biodiversity Data

**FIGURE 1:**
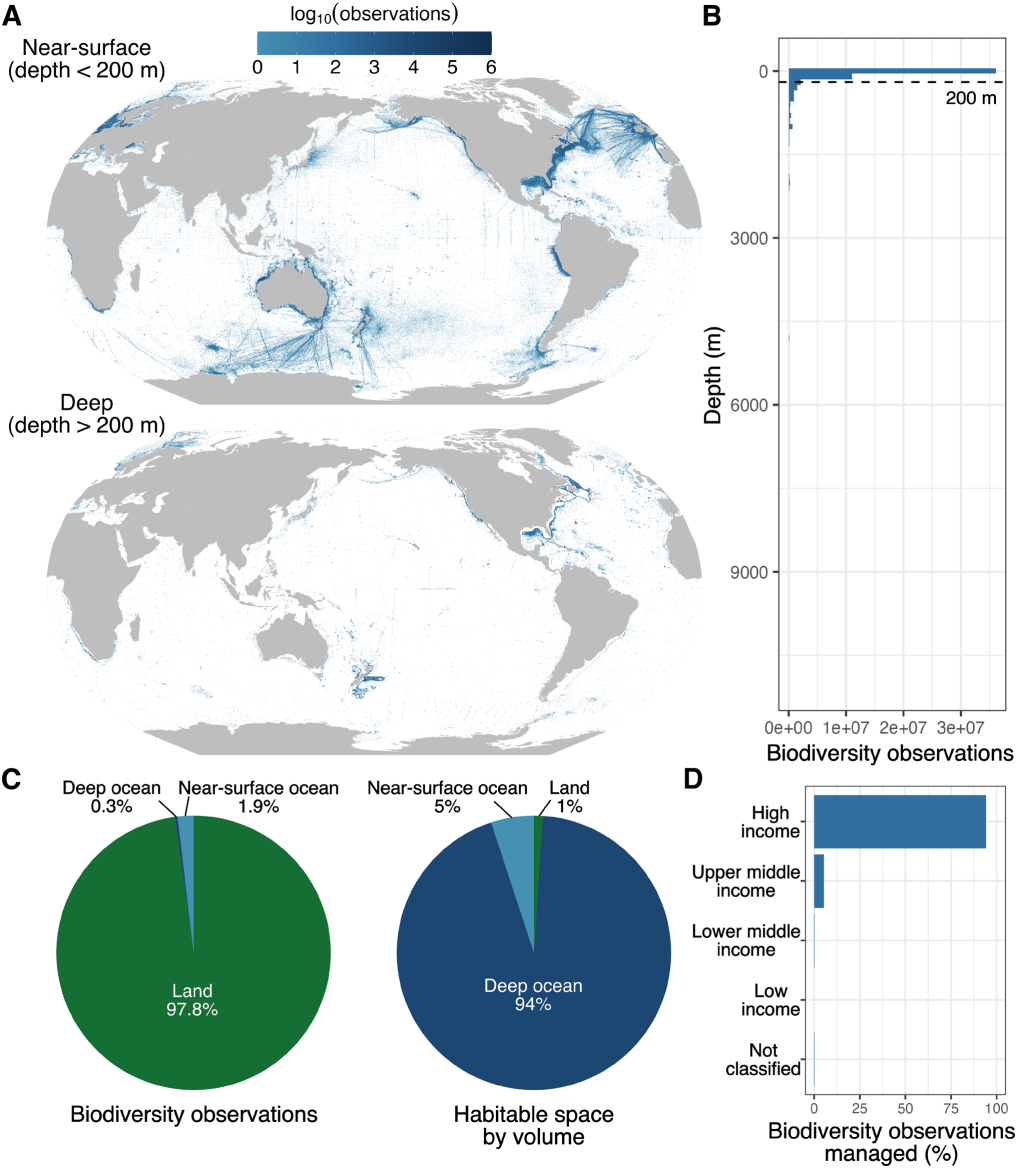
Gaps in ocean biodiversity data need to be filled to guide protection under the High Seas Treaty. (A) Maps of biodiversity observations in the Ocean Biodiversity Information System (OBIS 2024) reveal significant gaps in data coverage, particularly in the high seas. (B) Further data gaps are revealed in the ocean's critical third dimension, with a paucity of publicly accessible biodiversity data for the deep ocean below the photic zone (> 200 m depth). (C) Biodiversity data volume in the global ocean is dwarfed by similar data available in terrestrial ecosystems (from the Global Biodiversity Information Facility (GBIF.org 2024)), despite oceanic ecosystems representing the vast majority of habitable space on Earth. (D) Ocean biodiversity data which can guide protection of Earth's greatest commons are disproportionately managed by high income nations.

In terrestrial ecosystems, great effort has been directed toward addressing the non‐uniformity of biodiversity data to support conservation decision making (Chapman et al. 2024; Gonzalez et al. 2023). Yet the non‐uniformity and gaps are comparatively extreme in ocean ecosystems, and critically extend into the third dimension of depth (Figure 1). Area‐based protections often fail to account for biodiversity distribution along this third dimension, despite the deep ocean being home to Earth's largest animal communities (Jacquemont et al. 2024).

We now have a policy mechanism (pending ratification) to establish MPAs in the high seas (United Nations 2023). Algorithmic tools can aid in parsing diverse, voluminous data to make data‐informed recommendations about where to implement these protections (Sala et al. 2021), yet are biased not only by the values encoded in their objective functions but also deficiencies in the data on which they operate (Chapman et al. 2021). Thus, robust and publicly accessible biodiversity data is now a key constraint on designing and implementing effective and equitable protections under the mechanism afforded by the HST.

## 2 Data Gaps and Conservation Decisions

The protection mechanism afforded by the HST not only creates protected areas, but also delineates de facto areas of non‐protection toward which growing industrial activity will be directed. MPA proposals based on the ‘best available science and scientific information’ (United Nations 2023) are likely to be biased toward areas for which biodiversity observations are accessible. Other areas rich in biodiversity, but for which publicly accessible data is lacking, may become targets for industrial activity.

Historical cases of marine spatial planning underscore the critical role of biodiversity data in effective conservation planning. Consider the establishment of the Monterey Bay National Marine Sanctuary in the United States in 1992: this protected area, centred on the Monterey Submarine Canyon, originally excluded the adjacent Davidson Seamount. The extraordinary deep‐sea biodiversity of Davidson Seamount was not recognised at the time of the Sanctuary's designation, but became apparent with subsequent data availability. The Sanctuary boundaries were amended to include Davidson Seamount in 2008, but this case raises important questions: what would have happened in the intervening years if deep‐sea mining or offshore energy infrastructure had been technologically feasible at the time?

Similar decision‐making processes will soon occur throughout our global ocean. Knowledge (including biodiversity data) is an important source of power in environmental governance (Bennett 2019), particularly in spaces such as the open and deep ocean which are a relative ‘blank slate’ for policy (Schadeberg et al. 2023). The specific processes by which signatories interact to design, delineate and enforce protections under the HST remain to be seen, but biodiversity data represent an invaluable knowledge resource for creating narratives for protection based on the ‘best available science and scientific information’ (United Nations 2023). Ensuring equity of access to this knowledge‐based power (Crosman et al. 2022) requires that such biodiversity data is publicly accessible as a basis for signatories' proposed protections under the HST.

## 3 Collective Science Action

**FIGURE 2:**
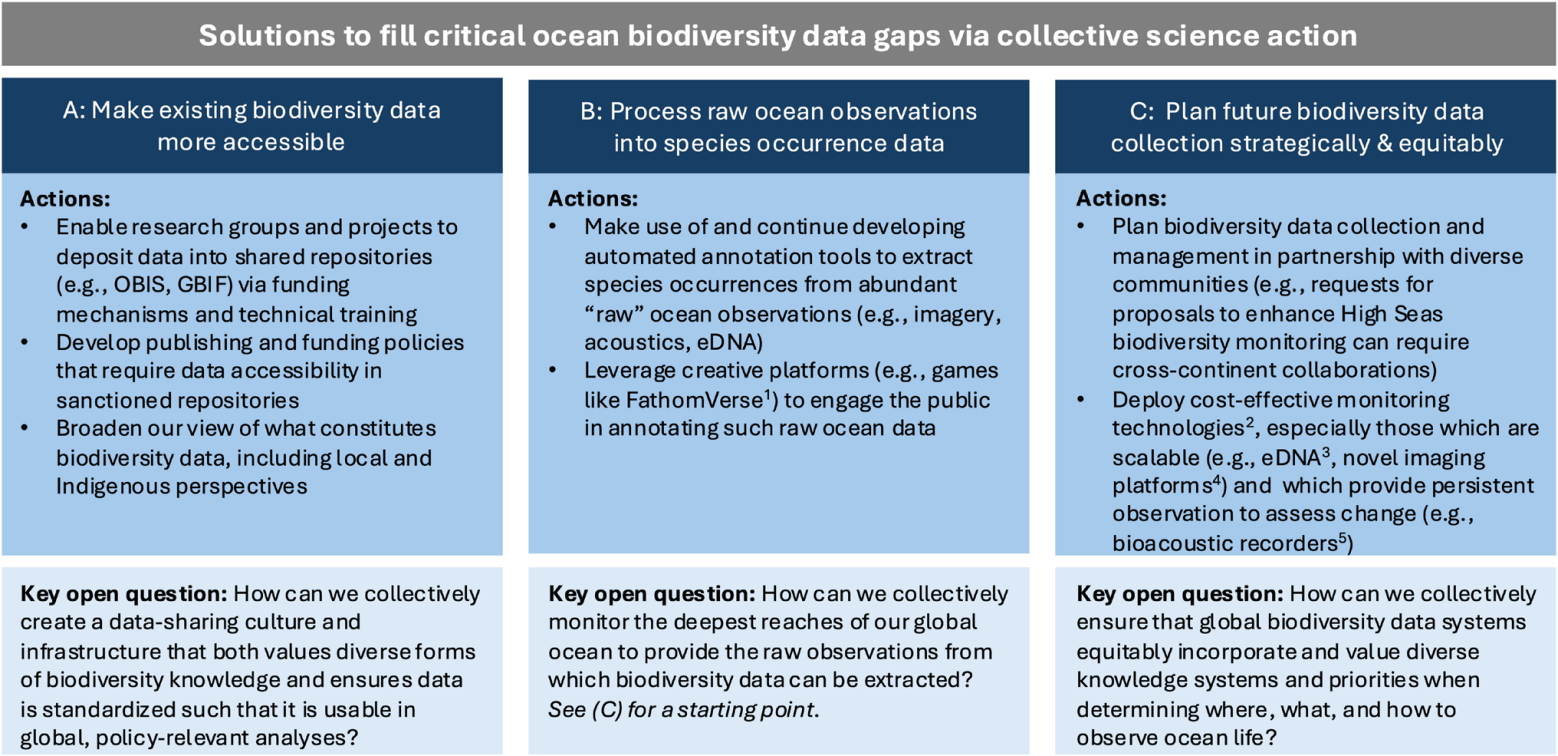
Solutions to fill critical ocean biodiversity data gaps via collective science action. For each solution (A–C), specific and practical actions that can be taken to realise these solutions are presented. Key open questions about how to implement these actions are also presented for each solution. ^1^Katija et al. (2022); ^2^Bell et al. (2023); ^3^Berry et al. (2021); ^4^Novy et al. (2022); ^5^Oestreich et al. (2024).

Current data disparities and the proximity of policy action under the HST make an important point clear: as a global community, we must take immediate and sustained collective action to provide biodiversity data to inform global ocean protections from the surface to the seafloor. The lack of biodiversity data for much of the global ocean is not exclusively because these ocean swaths have never been observed, but rather partly because observations have not been processed and/or made publicly accessible through open repositories (e.g., the Ocean Biodiversity Information System (OBIS); the Global Biodiversity Information Facility (GBIF)). Here we articulate three calls for collective action which can provide solutions (Figure 2) to overcome these obstacles, thus enhancing the potential efficacy of area‐based protections under the HST.

First, a combination of policies and cultural shifts can help to move biodiversity data sources along the spectrum from ‘private and bespoke’ (e.g., supplementary tables, internal lab hard drives) to ‘accessible and standardized’ (e.g., OBIS, GBIF). Although data at the former end is more difficult to access and synthesise, it is no less valuable and may, in aggregate, contain a greater volume and variety of data than global repositories (Heidorn 2008). Individual research groups can contribute to this solution immediately by depositing existing oceanic biodiversity data in accessible and standardised repositories. Creating a field‐wide movement to make disaggregated data accessible is a universal challenge in science, but there are proven, effective solutions. For example, molecular biologists *expect* to deposit new sequence data in an accessible and standardised database (i.e., GenBank) as a prerequisite for publishing, a norm resulting from coordination between GenBank's managers and journal editors (Cinkosky et al. 1991). A clearly articulated biodiversity data sharing policy coordinated across journals, requiring deposition in a sanctioned repository (e.g., GBIF, OBIS) is an important component of this data accessibility solution (Gomes et al. 2022; Figure 2A). Implementing strict journal policies without complementary educational, computational and financial resources risks placing undue and inequitable burdens on researchers. As a complementary policy, funders could support and require data deposition into global repositories (Dumanis et al. 2023). Building a culture of data sharing also necessitates broadening our notion of what constitutes biodiversity data. The historical emphasis on species observations is a reasonable starting point but comes with its own biases (Record and Vera 2021). Bringing new data sharers into the fold requires reflection on how we understand biodiversity—including relationships among species, local and Indigenous knowledge, ecological histories of locations and the ways that humans fit into open and deep‐ocean ecosystems.

We can also devise creative solutions to process existing ‘raw’ ocean observations (e.g., imagery, acoustics, eDNA) of ocean life into biodiversity data (Figure 2B). The power of participatory science (Bonney et al. 2014) represents a promising pathway to solutions across ocean‐observing modalities. The majority of publicly accessible terrestrial biodiversity observations are generated via participatory science (Chandler et al. 2017), yet these platforms engage people primarily where they live: on land. Few humans regularly interact directly with the open and deep ocean, so what role can participatory science play in observing its biodiversity? Nascent efforts aim to bring ocean observing to humans instead. For example, FathomVerse (Katija et al. 2022) is an interactive game through which participants classify biodiversity in unlabeled, real‐world deep‐ocean imagery. This approach could be similarly adopted for a diversity of data modalities. By appealing to our innate fascination with ocean life, creatively deployed participatory science platforms can play a key role in humanity's collective effort to observe the biodiversity of our ocean planet.

These first two solutions—building a culture of data accessibility and facilitating existing data processing—will help to increase volume, but alone might not address the three‐dimensional coverage bias in accessible biodiversity data. Filling the significant remaining data gaps is an enormous undertaking. Yet diverse knowledge of oceanic biodiversity and increasingly cost‐effective, accessible monitoring technologies provide solutions to strategically and equitably fill remaining data gaps (Figure 2C).

This third solution is not just about filling in the map: decisions about where and what to observe have downstream impacts on where and what we choose to protect. The vast majority of publicly accessible ocean biodiversity data come from the global north (Figure 1A) and are managed by high‐income nations (Figure 1D). To mitigate the risk of propagating these biases into proposed conservation actions (Chapman et al. 2021; Record et al. 2025), data collection and management programmes must be planned with research partners across our global community. Nations and communities around the globe have diverse knowledge of and priorities for ocean biodiversity observation and protection (Mulalap et al. 2020), which should be reflected in monitoring investments and data infrastructures. These diverse knowledge sources can help prioritise areas for further biodiversity data collection, and represent critical information in their own right for devising protections (Mulalap et al. 2020). Cost‐efficient biodiversity monitoring tools (e.g., eDNA, bioacoustics, imagery), deployed not only via vessels but also via global fleets of autonomous platforms (Johnson et al. 2022; Lehman 2018) will also be a critical piece of this solution (Figure 2C). Making such observations, past and future, publicly accessible can facilitate coordinated and strategic monitoring strategies, reducing costs and the potential for unnecessary redundancy in observations.

This is not to suggest that *all* biodiversity data should be made publicly accessible: whereas digitization and public sharing of knowledge can enable protection of biodiversity under the HST, it can also create risk of exploitation of culturally and economically valuable biodiversity and data. The federated architectures of data repositories (i.e., regional, country or community‐specific nodes which manage portions of the broader, decentralised database) can alleviate some of this risk, by affording communities agency over which data they make publicly accessible, and at what resolution. If conducted thoughtfully, we can collectively enhance both the geographic coverage of accessible biodiversity observation as well as the diversity of knowledge and priorities represented by the communities that collect and manage these data.

## 4 Conclusion

The High Seas Treaty is creating an opportunity for humanity to protect our greatest global commons. Although reliance on protected areas alone is a flawed approach (Levis et al. 2024), the area‐based protection mechanism afforded by the HST still represents a valuable tool for ocean biodiversity conservation. Realising this opportunity requires solutions that meaningfully and justly fill biodiversity data gaps throughout the open and deep sea. Collective science actions can provide solutions to achieve this goal (Figure 2). First, we can make existing oceanic biodiversity data publicly accessible through a combination of immediate action to deposit data in collectively held open repositories and field‐wide shifts in publishing and funding policies to require such data deposition in the future. Second, we can devise creative solutions to process raw ocean observations into invaluable biodiversity data. Whereas algorithmic tools are highly touted in this area, the power of participatory science should not be overlooked. Finally, future data collection efforts can be planned strategically to effectively, equitably and efficiently fill remaining gaps. Robust, publicly accessible biodiversity data is critical to ensuring that signatories can access the best available scientific information as a foundation for proposals for protection under the HST. Data alone does not ensure protection but is a key ingredient, providing information and associated authority toward implementing protections of the oceanic ecosystems on which our planet and society depend.

## Author Contributions

W.K.O. and M.S.C. conceived the paper, conducted the data analysis and visualisation and prepared the first draft; all authors contributed to refining the ideas and writing.

## Peer Review

The peer review history for this article is available at https://www.webofscience.com/api/gateway/wos/peer‐review/10.1111/ele.70168.

## Data Availability

All data and code are available at https://github.com/woestreich/ocean_biodiversity (https://doi.org/10.5281/zenodo.15662599).
